# Supplementary material for: Dismal pathological response to neoadjuvant chemotherapy in stage III breast cancer patients in Tanzania: A retrospective review
Source: PLoS One. 2025 Apr 23;20(4):e0321187. doi: 10.1371/journal.pone.0321187 (PMC12017560; doi:10.1371/journal.pone.0321187)
Supplement: Attachment — Submitted filename: Response to Reviewers.docx [file pone.0321187.s002.docx]

**Cover Letter**

**Journal Name: PLOS ONE**

**Date of Submission: 30^th^ January, 2025**

**Name of Corresponding Author: Nashivai Elias kivuyo**

**Contact Information of the Corresponding Author**

**Address: P.O. BOX 65001, Dar es Salaam, Tanzania.**

***Email: nashivaelias@gmail.com**

***Tel: +255 753448229**

***Article Type: Original Research**

**Dear Editor,**

**RE: Dismal Pathological Response to Neoadjuvant Therapy in Stage III Breast Cancer Patients in Tanzania: A Retrospective Review**

We would like to express our gratitude to the reviewers for their insightful comments and suggestions regarding our manuscript titled “Dismal Pathological Response to Neoadjuvant Therapy in Stage III Breast Cancer Patients in Tanzania: A Retrospective Review.” We appreciate the opportunity to address the reviewers' concerns, which have helped us improve our manuscript. Below, we outline our responses to each comment.

| **SNo.** | **Comments from the Reviewer** | PI’s response |
| --- | --- | --- |
| 1. | None of the patients received targeted therapy in the NAT setting. This is a retrospective study and without targeted treatments, we could not conclude the results. There is a selection bias in this cohort. | We appreciate your comment. In Tanzania, the use of targeted therapies in neoadjuvant settings is significantly limited due to cost constraints and resource availability. As such, targeted therapy is used in the adjuvant settings to a few patients who can afford. In our cohort, none of the patients received targeted therapy and we have discussed this as one of the contributing factors to the low response. |
| 2 | Conclusion: States that ‘a relatively poor pathological response was observed’, however a comparison is not provided? Poor compared to what? Western counterparts? Please specify. | We appreciate this comment. The conclusion has been revised to specify that the observed pathological response is poor compared to data from Western populations. |
| 3 | Line 69: ‘Despite the relatively low incidence of BC in Africa, mortality remains high, and overall survival rates are disproportionally lower in SSA’ - A comparison is not provided again. Relatively low incidence compared to what? Also, would be better to specify the incidence and overall survival rates for clarity rather than providing a generic statement. | We have clarified the comparison by noting that the incidence of breast cancer in Africa is relatively low compared to incidence rates in North America and Europe. |
| 4 | Please define the duration of neoadjuvant therapy (number of cycles and lines) | Thank you for your comments. We have included this in Table 1 of results. |
| 5 | Figure 2 is redundant and maybe safely omitted | We agree with your observation. We have removed Figure 2 from the manuscript. |
| 6 | Was a modified radical mastectomy (mastectomy + axillary lymph node dissection) performed in all patients without an attempt at sentinel lymph node biopsy/targeted axillary dissection? This may reflect a difference in management from Western populations where there is an emphasis on de-escalation of complete axillary dissection. | Sentinel lymph node (SLN) biopsy is not performed at our institution due to resource constraints and lack of expertise in our setting, which differ from practices in Western populations. |
| 7 | Do the authors have data regarding completion of planned chemotherapy cycles? This is an important covariate and should be compared between the two study groups. | Breast cancer care in our setting is provided between two hospitals, the ocean road cancer institute where patients receive chemotherapy and radiotherapy and Muhimbili National Hospitals where surgery for breast cancer is performed. When patients start NAT, they will only be sent for surgery after they have completed the planned NAT. Hence, all patients included in our study had completed their planned chemotherapy cycles. This is why only n181 patients were analyzed despite 376 starting NAT. If you refer in figure 1, 157 patients were not sent back for surgery despite starting NAT. The possible reason would include treatment abandonment or disease progression that would eliminate the role for surgery. We have included this in our discussion. |
| 8 | Do the authors have data regarding grade chemotherapy-related toxicities? While they document higher pCR rates with more aggressive neoadjuvant therapy (AC-T vs. AC or AT), it would be worthwhile to explore if this was at the expense of higher significant adverse rate. This may also be a reason for discontinuation of neoadjuvant treatment. | Due to the retrospective nature of this study, documentation of chemotherapy-related toxicities was suboptimal. We have acknowledged this limitation in the discussion and suggest that further prospective studies could better assess toxicity and its impact on treatment completion and outcomes. |
| 9 | Can the authors comment about the utilization of screening mammography in Tanzania? What proportion of the cases were screening detected? In the absence of widespread screening, tumors are more likely to present at advances stages, which might be possible in their cohort | In Tanzania mammography services are confined to only two tertiary hospitals serving over 60 million people. They are mainly used for diagnostic rather than screening. Currently, we don’t have a national screening program using mammography. |
| 10 | Lines 257-268: In addition to recommending strategies for early detection, can the authors comment about measures to make modern neoadjuvant therapies available? For example, immunotherapy (Keynote 522 regimen) has been incorporated as the standard of care for neoadjuvant treatment of TNBC. Are there any measures which may be undertaken at a systems level to make these available in a LMIC setting? | We agree with your comments, however immunotherapies are very expensive and not yet readily available in Tanzania. The harmonized guideline for breast cancer management in SSA also recognizes this limitation and does not include them in routine standard of care in SSA. However, we agree that there is a need for improvement, such as advocating for systemic changes in healthcare infrastructure and developing local partnerships to enhance treatment availability in low-to-middle-income countries. |

Thank you for considering our manuscript for publication. We look forward to the opportunity to contribute to the academic dialogue on breast cancer treatment in Tanzania and potentially improve outcomes for our patients.

Yours sincerely,

Nashivai Kivuyo

Corresponding Author
